# In Vitro Analysis of the Dynamic Role of the Bacterial Virulence Factors in Skin Wound Healing

**DOI:** 10.3390/ijms262110472

**Published:** 2025-10-28

**Authors:** Ayat S Hammad, Sarah H. Zahedy, Shatha S. Elqasass, Sawsan Sudqi Said, Abdelrahman M. Elgamal, Nouf N Mahmoud, Maha Al-Asmakh

**Affiliations:** 1 Department of Biomedical Sciences, College of Health Sciences, QU Health, Qatar University, Doha 2713, Qatars.said@qu.edu.qa (S.S.S.); abdulrahman.elgamal@qu.edu.qa (A.M.E.);; 2 Biomedical Research Center, Qatar University, Doha 2713, Qatar; 3 Faculty of Pharmacy, Al-Zaytoonah University of Jordan, Amman 11733, Jordan

**Keywords:** wound healing, skin, virulence factors, bacteria, inflammatory markers

## Abstract

The skin acts as a primary barrier against environmental insults and maintains homeostasis. Injury initiates a wound healing cascade of hemostasis, inflammation, proliferation, and remodeling. In chronic wounds, persistent microbial colonization and inflammation disrupt this process, with bacterial virulence factors acting as key drivers. While the microbiome’s role in chronic wounds is recognized, the effects of individual virulence factors on acute repair remain unclear. Therefore, this study investigated the differential effects of virulence factors derived from five skin-associated bacterial species on acute wound healing dynamics. In this context, virulence factors from *Pseudomonas aeruginosa*, *Staphylococcus aureus*, *Streptococcus pyogenes*, *Lactobacillus plantarum*, and *Enterococcus faecalis* were tested on HDF-n cell viability and wound closure, with multiplex ELISA used to assess inflammatory mediator secretion and underlying mechanisms. Overall, virulence factors were generally well tolerated across concentrations (0.05–16 µg/mL) and time points (24, 48 h), with cell viability >80%, except for *S. aureus*, which reduced viability to ~70% at higher concentrations by 48 h. Wound healing responses varied markedly: *S. aureus* significantly impaired closure in a dose-dependent manner (~10% closure at 16 µg/µL, 48 h), and *E. faecalis* similarly delayed repair. In contrast, factors from *P. aeruginosa*, *S. pyogenes*, and *L. plantarum* showed neutral or mildly pro-healing effects. Notably, co-treatment with *S. pyogenes* partially rescued impairment caused by *S. aureus* and *E. faecalis*. Collectively, these findings highlight that bacterial virulence factors have variable impacts on acute wound healing. These findings suggest potential therapeutic applications through targeted modulation or combinations of bacterial factors.

## 1. Introduction

Skin, the largest organ of the human body, serves as a dynamic barrier that protects against chemical insults and pathogenic organisms while maintaining hydration, regulating temperature, synthesizing vitamin D, and supporting excretory functions [1,2]. As such, extensive damage to the skin not only disrupts these functions but also poses a grave threat to life [1,2]. Consequently, extensive skin injury disrupts these essential functions and poses a serious threat to survival [1,2]. Wound healing is a highly coordinated, multifaceted biological process that restores skin integrity through a cascade of cellular activities and molecular signaling networks [1,2,3]. This process involves the tightly regulated interplay of immune cells, cytokines, and growth factors that collectively drive tissue regeneration [1,2,3]. Skin wounds are broadly categorized as acute or chronic based on their etiology and healing dynamics [1,2]. Chronic wounds are defined by their failure to progress through the normal healing trajectory and are typically associated with prolonged inflammation, persistent infection, and extensive tissue necrosis [4]. In contrast, acute wounds follow a temporally ordered sequence of hemostasis, inflammation, proliferation, and remodeling, each governed by distinct cellular and molecular events [5].

During hemostasis, activated platelets rapidly form a clot and release critical growth factors, including platelet-derived growth factor (PDGF), transforming growth factors (TGFs), vascular endothelial growth factor (VEGF), and fibroblast growth factors (FGFs), which initiate the repair process [6,7]. This is followed by the inflammatory phase, wherein innate immune cells eliminate invading pathogens and prepare the wound bed for repair [8,9]. This stage is characterized by the recruitment of neutrophils and pro-inflammatory macrophages, which secrete cytokines such as tumor necrosis factor-alpha (TNF-α) and interleukin-1 (IL-1) to amplify immune cell recruitment [7]. Subsequently, the proliferative phase ensues, characterized by granulation tissue formation, neovascularization, and re-epithelialization, facilitated by a pro-healing milieu of T regulatory cells and M2 macrophages [2,10]. The final remodeling phase involves the maturation of granulation tissue into scar tissue, with a concurrent decrease in immune cell presence through apoptosis or migration [10,11]. Disruption of this complex, phased process results in non-resolving inflammation and impaired healing [12].

Impaired wound healing represents a major clinical and economic burden, affecting millions of patients and costing billions of dollars annually in the United States alone [13]. Several intrinsic and extrinsic factors—including aging, medications, immunodeficiency, diabetes, neuropathy, and vascular insufficiency—are known to impede healing [14]. Additionally, the skin microbiome has emerged as a pivotal determinant of wound outcomes, exerting both beneficial and detrimental effects through its modulation of infection and inflammation [15,16,17,18]. Recent evidence implicates bacterial virulence factors as key mediators that can disrupt immune regulation and derail normal tissue repair [19,20,21,22].

However, most studies investigating wound microbiota have focused on chronic wounds, often examining either single bacterial strains or polymicrobial consortia derived from clinical samples or diabetic wound animal models [15,23,24,25]. Given the diverse microbial communities that colonize healthy skin and the profound influence of the early wound microbiome on the transition from acute to chronic states [23,26,27,28,29], it is critical to examine their role during the acute phase of healing.

Understanding how individual virulence factors modulate host responses could elucidate the mechanisms by which certain bacteria exacerbate chronicity or, conversely, promote tissue repair. Therefore, this study aims to systematically investigate the effects of virulence factors from both commensal and pathogenic skin-associated bacteria on acute wound healing, with a focus on their influence on inflammatory signaling. Such insights could enable the development of targeted microbial-based therapeutics and predictive biomarkers for individuals at risk of chronic wound development, ultimately advancing precision wound care and improving patient outcomes.

## 2. Results

### 2.1. Bacterial Culture and Quantification of Virulence Factor Secretion

All bacterial strains—*P. aeruginosa*, *S. aureus*, *S. pyogenes*, *L. plantarum*, and *E. faecalis*- were cultured for 24 h to reach the stationary phase, which was verified by optical density (OD_600_) readings (Supplementary Table S1). Culture supernatants were harvested, filtered, and subjected to protein quantification using the BCA. Detectable levels of secreted proteins were present in all supernatants (Supplementary Table S2), confirming their suitability for downstream assays. Protein concentrations varied between strains, indicating distinct secretion profiles.

### 2.2. Cell Viability Assessment

To elucidate the impact of diverse bacterial virulence factors on cellular viability, the alamarBlue assay was utilized. Prior to the experimental design, an extensive literature review was undertaken to determine suitable concentration parameters for the virulence factors. However, no prior studies were identified that directly investigated the effects of these factors on HDF-n or similar cell types. This gap in the existing literature necessitated the establishment of an experimental concentration range. Accordingly, virulence factors were tested across a range of concentrations from 0.05 µg/mL to 16 µg/mL. Specific concentrations of 0.05, 1, 3, 5, 12, and 16 µg/mL were assessed at two critical time intervals—24 and 48 h post-exposure—to discern any temporal variations in cellular response.

**Figure 1:**
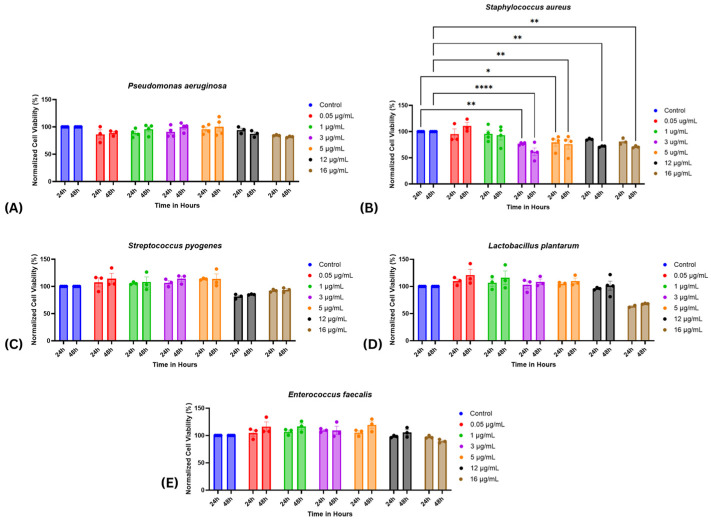
Cell Viability of Various Bacterial Virulence Factors. This figure presents the normalized cell viability of cells exposed to increasing concentrations (0.05, 1, 3, 5, 12, 16 µg/mL) of supernatants from different bacterial strains over 24 and 48 h. Strains include (**A**): *P. aeruginosa*, (**B**): *S. aureus*, (**C**): *S. pyogenes*, (**D**): *L. plantarum*, (**E**): *E. faecalis*. Data are expressed as means ± SEM from 3 to 4 independent experiments. Statistical significance was determined using two-way ANOVA with Fisher’s post hoc test. Statistical significance was observed only for S. aureus * *p* < 0.05, ** *p* < 0.01, **** *p* < 0.0001 vs. control.

Our findings revealed that *P. aeruginosa* was well-tolerated by the HDF-n cells, maintaining approximately 80% viability at the highest concentration tested (16 µg/mL) after 48 h, indicating significant cellular resilience Figure 1A. Conversely, *S. aureus* induced a pronounced dose-dependent cytotoxic effect, evident from concentrations as low as 3 µg/mL beginning at 24 h post-treatment. Viability markedly decreased with escalating concentrations, dropping to about 70% at 16 µg/mL by the 48-hour mark Figure 1B. This data suggests that concentrations of 5 and 12 µg/mL, which maintained cell viability at 79% and 75% at 24 h and 85% and 72% at 48 h, respectively, may be optimal for further investigation using *S. aureus* due to their balance between measurable cellular response and viability maintenance.

On the other hand, *S. pyogenes* exhibited high tolerance across all tested concentrations, consistently showing cell viability above 80% at all time points Figure 1C. Similarly, *L. plantarum* also demonstrated well-tolerated concentrations up to 12 µg/mL with cell viability remaining above 90%; however, this decreased significantly at 16 µg/mL to the 60% range in both assessed time points Figure 1E. This significant reduction in viability suggests a limit to the concentration of *L. plantarum* that HDF-n cells can tolerate without substantial cytotoxic effects. Lastly, *E. faecalis* exhibited minimal cytotoxic effects, maintaining cell viability around 90% even at the highest concentration tested Figure 1E.

### 2.3. Wound Healing Assay

**Figure 2:**
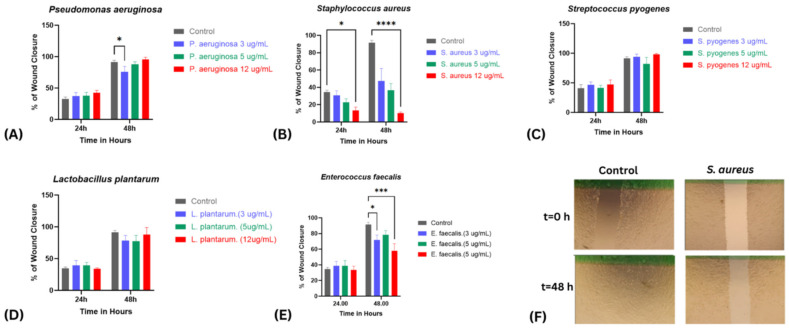
Effects of Bacterial Virulence Factor Supernatants on Fibroblast-mediated Wound Closure. HDF-n monolayers were scratched to simulate wounds and treated with bacterial supernatants (3, 5, 12 µg/mL). Wound closure was monitored for 24–48 h. (**A**) *P. aeruginosa* (**B**) *S. aureus* (**C**) *S. pyogenes* (**D**) *L. plantarum* (**E**) *E. faecalis* (**F**) Representative phase-contrast micrographs showing wound closure in untreated control versus S. aureus–treated monolayers at 0 and 48 h, highlighting the inhibitory effect of *S. aureus* on fibroblast migration. Data are presented as mean ± SEM from three independent experiments. Data are presented as mean ± SEM from three independent experiments. Statistical analysis was performed using two-way ANOVA with post hoc tests. *p* values are indicated as follows: *p* < 0.05 (*), *p* < 0.001 (***), *p* < 0.0001 (****).

The impact of bacterial virulence factors on fibroblast-mediated wound closure was assessed using a scratch assay across concentrations of 3, 5, and 12 µg/mL over 24–48 h (Figure 2A–E). Distinct strain-specific patterns emerged, revealing variable influences on cell migration and tissue repair dynamics. *P. aeruginosa* supernatants did not significantly alter wound closure at any concentration after 24 h. By 48 h, a modest but statistically significant reduction in closure was observed at 3 µg/mL compared with the control (*p* < 0.05). However, this effect was not sustained at higher concentrations, as wound closure at 5 and 12 µg/mL recovered to levels comparable to the control (~70–80% closure by 48 h), indicating that the inhibitory effect at 3 µg/mL was transient and not dose-dependent (Figure 2A). Similarly, *L. plantarum* supernatants showed no change in wound closure at any concentration at 24 h. By 48 h, a mild, non-significant reduction was observed at 3 and 5 µg/mL, whereas treatment with 12 µg/mL restored closure to levels comparable to the control, suggesting that the slight inhibition seen at lower concentrations was transient and reversed at higher concentrations (Figure 2E).

In contrast, *S. aureus* supernatants induced a pronounced, dose- and time-dependent inhibition of wound repair, detectable as early as 24 h and becoming markedly more evident by 48 h. At 24 h, cells treated with 12 µg/mL exhibited ~20% less wound closure compared with the control. By 48 h, this inhibitory effect intensified in a clear dose-dependent manner, with wound closure reduced to approximately 47%, 36%, and 10% at 3, 5, and 12 µg/mL, respectively, representing up to an 80% reduction relative to the control group (Figure 2B). Similarly, *E. faecalis* caused a marked, concentration-dependent delay, reducing wound closure to ~71% at 3 µg/mL and ~57% at 12 µg/mL after 48 h (Figure 2C).

**Figure 3:**
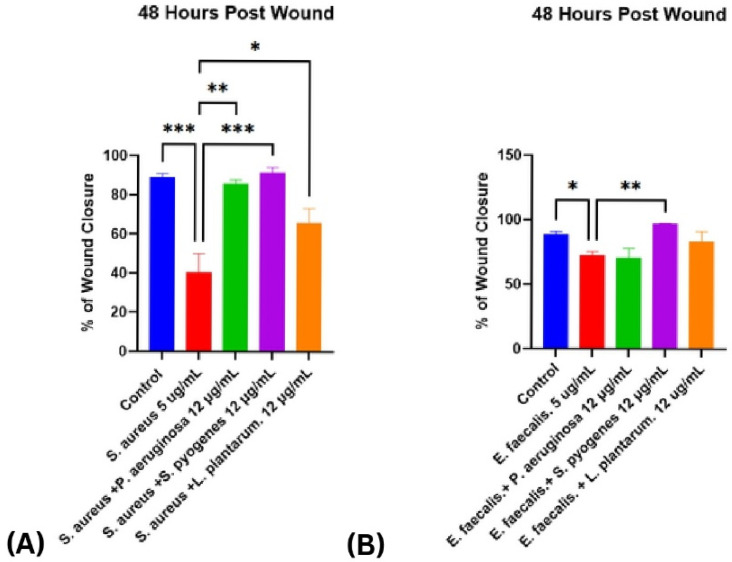
Co-treatment with Selected Bacterial Virulence Factors Reverses the Inhibitory Effects of *S. aureus* and *E. faecalis* on Fibroblast Wound Closure. (**A**) Percentage of wound closure at 48 h post-scratch in HDF-n monolayers treated with *S. aureus* virulence factors alone (5 µg/mL) or in combination with *P. aeruginosa*, *S. pyogenes*, or *L. plantarum* (12 µg/mL each). (**B**) Percentage of wound closure in HDF-n monolayers treated with *E. faecalis* virulence factors alone (5 µg/mL) or in combination with *P. aeruginosa*, *S. pyogenes*, or *L. plantarum* (12 µg/mL each). Data are presented as mean ± SEM from 2 to 3 independent experiments. Statistical significance was determined using two-way ANOVA. followed by Fisher’s least significant difference test. *p* < 0.05 (*), *p* < 0.01 (**), and *p* < 0.001 (***) were considered significant.

Conversely, *S. pyogenes* supernatants demonstrated wound closure rates that were comparable to, and in some cases slightly higher than, the control group across all tested concentrations. At 24 and 33 h, cells treated with 3, 5, and 12 µg/mL showed progressive closure similar to controls, and by 48 h closure reached ~90–100% across all groups. No statistically significant differences were detected, but the consistently equal or slightly enhanced closure suggests a potentially beneficial effect on fibroblast migration and wound repair, which could become more pronounced at higher concentrations than those tested (Figure 2D). Interestingly, co-treatment experiments demonstrated that the pronounced delay in wound healing caused by *S. aureus* was substantially mitigated when combined with *P. aeruginosa*, *S. pyogenes*, or *L. plantarum* (Figure 3A), while the inhibitory effect of *E. faecalis* was similarly reversed upon co-treatment with *S. pyogenes* (Figure 3B). These findings highlight a striking contrast between the detrimental effects of *S. aureus* and *E. faecalis* and the neutral or even protective influences of *P. aeruginosa*, *L. plantarum*, and *S. pyogenes*, suggesting that interspecies interactions can substantially modulate the outcome of wound repair.

### 2.4. Modulatory Effects of Bacterial Virulence Factors on Cytokine Release from Stimulated Fibroblasts

**Figure 4:**
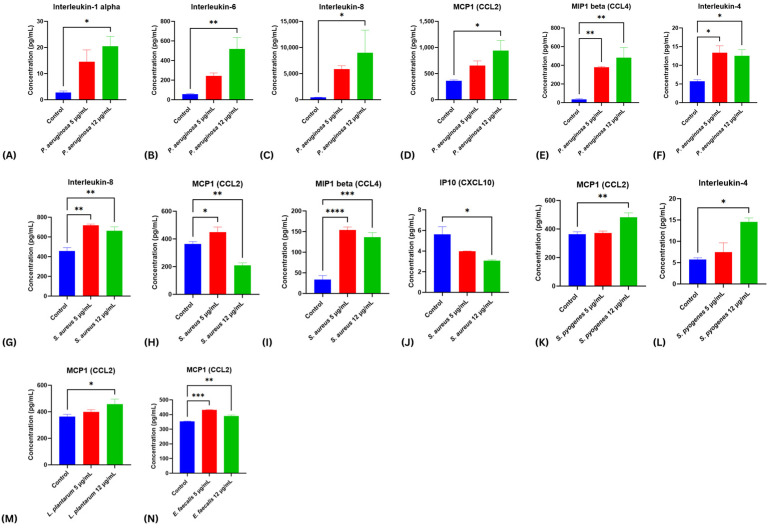
Modulation of cytokine secretion in HDF-n fibroblasts following exposure to bacterial virulence factor supernatants. HDF-n cultures were subjected to scratch injury and treated with bacterial supernatants at 5 and 12 µg/mL for 48 h. Cytokine and chemokine concentrations were quantified using a multiplex immunoassay. (**A**–**F**) *P. aeruginosa* induced significant increases in IL-1α, IL-6, IL-8, MCP-1 (CCL2), MIP-1β (CCL4), and IL-4, with a dose-dependent trend. (**G**–**J**) *S. aureus* treatment elevated IL-8 and MIP-1β while reducing MCP-1 and IP-10 (CXCL10). (**K**,**L**) *S. pyogenes* significantly enhanced MCP-1, and IL-4. (**M**) *L. plantarum* induced a modest but significant increase in MCP-1 at the highest concentration. (**N**) *E. faecalis* increased MCP-1 secretion. Data represent means ± SEM of two independent experiments. Statistical significance was determined using two-way ANOVA with post hoc tests; *p* < 0.05 (*), *p* < 0.01 (**), *p* < 0.001 (***), *p* < 0.0001 (****) compared with untreated controls.

Cytokine secretion profiles were assessed in HDF-n fibroblasts at 48 h post-scratch following exposure to bacterial virulence factor supernatants at 5 and 12 µg/mL (Figure 4A–M). Distinct strain-specific immune signatures were observed, reflecting the differential wound-healing phenotypes identified in scratch assays.

Exposure to *P. aeruginosa* supernatants elicited a broad, dose-dependent pro-inflammatory response across multiple cytokines. IL-1α increased to ~20 pg/mL at 12 µg/mL (*p* < 0.05), IL-6 rose sharply to ~520 pg/mL (*p* < 0.01), and IL-8 secretion escalated dramatically from baseline to nearly 12,000 pg/mL (*p* < 0.05) (Figure 4A–C). MCP-1 (CCL2) also increased to ~930 pg/mL (*p* < 0.05), while MIP-1β (CCL4) rose steeply from undetectable levels to >480 pg/mL (*p* < 0.01) (Figure 4D,E). IL-4 doubled from ~5 pg/mL in controls to ~12 pg/mL at both concentrations (*p* < 0.05) (Figure 4F). ICAM-1 displayed a dose-dependent increase, although this did not reach statistical significance, while IP-10 (CXCL10) showed a mild, non-significant reduction (Supplementary Figure S1). Together, these findings indicate that *P. aeruginosa* supernatants drive a strong pro-inflammatory milieu dominated by IL-6, IL-8, and MIP-1β, consistent with a robust but non-detrimental inflammatory activation, in line with the neutral wound-healing phenotype observed.

By contrast, *S. aureus* supernatants elicited a cytokine signature that closely paralleled its pronounced inhibitory effect on fibroblast migration and wound closure. IL-8 secretion was significantly elevated at both 5 and 12 µg/mL (*p* < 0.01), highlighting a sustained pro-inflammatory state (Figure 4G). MIP-1β (CCL4) levels were markedly increased, rising from <50 pg/mL in controls to >120 pg/mL at both concentrations (*p* < 0.001), while MCP-1 (CCL2) also showed a significant elevation at the highest concentration tested (Figure 4H,I). In contrast, IP-10 (CXCL10) was significantly reduced at 12 µg/mL (*p* < 0.05; Figure 4J), and no significant alterations were detected in ICAM-1, IL-1α, IL-6, or IL-4 (Supplementary Figure S2). This cytokine profile—characterized by excessive IL-8 and MIP-1β production, coupled with suppressed IP-10—suggests a shift toward a hyperinflammatory yet dysregulated environment, impairing coordinated chemokine signaling. Such an imbalance likely contributes to the severe inhibition of fibroblast migration and the delayed wound closure phenotype observed with *S. aureus*.

In comparison, *S. pyogenes* supernatants promoted a cytokine profile indicative of a pro-migratory and repair-supporting environment. At 12 µg/mL, significant increases in MCP-1 (CCL2), IL-4, and CXCL10 (*p* < 0.05) were observed, all of which are key mediators of fibroblast recruitment, re-epithelialization, and tissue remodeling. A dose-responsive trend toward increased MIP-1β secretion was also observed, although it was not statistically significant (Supplementary Figure S3). These immune signatures align with the enhanced wound closure phenotype observed in scratch assays, suggesting that *S. pyogenes* supports fibroblast-mediated repair through activation of chemokine- and cytokine-driven signaling.

Supernatants from *L. plantarum* had minimal impact on cytokine release, consistent with its neutral effect on wound healing. No significant changes were observed across ICAM-1, IL-6, IL-1α, IL-4, IL-8, CXCL10, or CCL4, though a small but statistically significant increase in MCP-1 (CCL2) was detected at 12 µg/mL, suggesting a limited chemokine-modulatory effect without broader inflammatory activation (Supplementary Figure S4).

Finally, *E. faecalis* supernatants were characterized by a selective and statistically significant increase in MCP-1 (CCL2) at 12 µg/mL (*p* < 0.01), while ICAM-1, IL-1α, and IL-6 remained unchanged (Supplementary Figure S5). This narrow cytokine response correlates with the delayed wound healing observed in scratch assays, suggesting that *E. faecalis* may impair tissue repair by driving localized MCP-1–mediated monocyte recruitment without triggering a broader inflammatory cascade.

## 3. Discussion

This study provides novel mechanistic insights into how secreted virulence factors from distinct skin-associated bacterial species modulate fibroblast-mediated wound repair. By integrating phenotypic wound closure assays with multiplex cytokine profiling, we demonstrate that the effects of bacterial secretomes on wound healing are highly strain-specific and governed by differential activation of inflammatory and chemokine pathways. These findings underscore the dynamic interplay between bacterial virulence, host immune responses, and tissue repair mechanisms, highlighting both pathogenic and potentially therapeutic microbial influences within the cutaneous microenvironment.

Exposure to *Pseudomonas aeruginosa* supernatants elicited a broad, dose-dependent pro-inflammatory cytokine response, marked by increased secretion of IL-1α, IL-6, IL-8, MCP-1 (CCL2), MIP-1β (CCL4), and IL-4. Interestingly, this heightened inflammatory activation did not translate into a corresponding inhibition of fibroblast migration or wound closure, suggesting that fibroblasts can tolerate or adapt to *P. aeruginosa* secretory products in the absence of highly cytotoxic virulence components. This observation aligns with previous studies showing that wild-type *P. aeruginosa* supernatants often do not impede wound closure in vitro, whereas quorum-sensing-deficient mutants significantly delay repair [30]. Quorum sensing, therefore, appears to be a major determinant of *P. aeruginosa* pathogenicity and represents a promising therapeutic target for modulating its behavior [30]. In contrast, purified virulence factors such as pseudolysin and protease IV have been shown to profoundly impair repair processes [19]. Clinically, the detection of *P. aeruginosa* in wounds does not consistently correlate with impaired healing outcomes [31,32], reflecting the context-dependent nature of its pathogenic potential. The variability in clinical outcomes likely stems from strain-specific virulence repertoires, the regulatory state of quorum-sensing systems, and the immunological landscape of the host. Collectively, these findings suggest that *P. aeruginosa* contributes to wound pathology in a conditional manner, and that elucidating strain-dependent virulence mechanisms could enable the development of targeted anti-virulence or quorum-modulating therapies aimed at mitigating tissue damage while preserving commensal tolerance.

In contrast, *Staphylococcus aureus* exhibited a potent and consistent inhibitory effect on fibroblast migration and wound closure, with nearly 80% reduction observed at higher concentrations. This pronounced impairment was associated with a cytokine profile dominated by elevated IL-8 and MIP-1β levels, suppression of IP-10, and decreased MCP-1 expression—features that collectively indicate a dysregulated, hyper-inflammatory milieu that promotes leukocyte recruitment but fails to resolve inflammation. Such a pattern closely mirrors clinical findings in non-healing wounds, where sustained IL-8 overexpression correlates with delayed tissue regeneration [33]. The pathogenic activity of *S. aureus* is mediated by a repertoire of virulence determinants—including TSST-1, leukocidins, enterotoxins, and exfoliatins—that disrupt host cell membranes, degrade extracellular matrix components, and perpetuate inflammatory signaling [21,34,35,36]. These multifactorial interactions plausibly explain the severe inhibition of fibroblast-driven closure observed in our model. Furthermore, the increasing prevalence of methicillin-resistant *S. aureus* (MRSA) strains exacerbates these effects, as resistant isolates often express enhanced virulence and elicit prolonged inflammatory responses that further compromise wound resolution [37,38,39,40]. Together, these data reinforce *S. aureus* as a principal driver of chronic wound pathogenesis, wherein unbalanced cytokine signaling and persistent immune activation undermine tissue repair and foster wound persistence.

Unlike *S. aureus*, *Streptococcus pyogenes* exhibited a reparative profile, promoting fibroblast migration and wound closure comparable to—or slightly exceeding—control levels. This pro-healing phenotype was accompanied by increased secretion of MCP-1 (CCL2) and IL-4, along with moderate elevations in MIP-1β and CXCL10, cytokines that orchestrate monocyte recruitment, angiogenesis, and epithelial regeneration. The capacity of *S. pyogenes* to modulate inflammatory responses without inducing cytotoxicity may explain the typically mild and self-limiting nature of its superficial infections [41]. Its principal virulence determinants—including lipoteichoic acids, M protein, opacity factor, streptolysins O and S, and streptococcal pyrogenic exotoxins—are known to influence immune activation and tissue remodeling in a context-dependent manner [42]. The late increase in CCL2 and CXCL10 observed here suggests not only the initiation of inflammatory recruitment but also the activation of re-epithelialization and tissue remodeling pathways. The concurrent elevation of IL-4 further supports a shift toward resolution and regeneration, facilitating the transition from the inflammatory to the proliferative phase of healing. Notably, co-treatment experiments revealed that *S. pyogenes* supernatants partially reversed the inhibitory effects of *S. aureus* and *E. faecalis*, suggesting that it exerts a modulatory or antagonistic influence within polymicrobial environments. This observation underscores the importance of interspecies interactions in shaping wound outcomes, implying that certain bacterial species can mitigate pathogenic effects by rebalancing the local immune milieu.

*Lactobacillus plantarum* supernatants displayed a neutral to mildly beneficial influence on fibroblast migration, consistent with its established probiotic and immunomodulatory properties. The cytokine profiles remained largely unaltered, except for a modest but significant increase in MCP-1 (CCL2) at higher concentrations, reflecting a limited yet physiologically favorable activation of chemokine signaling. These results concur with prior studies demonstrating that *L. plantarum* enhances keratinocyte migration, stimulates collagen deposition, and reduces bacterial burden while modulating IL-8-mediated inflammation [43,44,45]. Furthermore, *L. plantarum* has been shown to interfere with *P. aeruginosa* virulence gene expression and suppress its pathogenic activity [20,46,47], thereby promoting a more balanced microbial environment. The dual antimicrobial and anti-inflammatory capacities of *L. plantarum* position it as a strong candidate for microbiome-based wound therapeutics, particularly in the context of antibiotic resistance and chronic infections. By simultaneously curbing pathogen virulence and supporting host regeneration, *L. plantarum* exemplifies the therapeutic potential of beneficial microbes in restoring wound homeostasis [48,49].

Conversely, *Enterococcus faecalis* was consistently associated with delayed wound closure in a concentration-dependent manner, coupled with selective upregulation of MCP-1 (CCL2) and minimal alteration of other cytokines. This restricted inflammatory response suggests that *E. faecalis* drives localized, unresolved inflammation characterized by monocyte recruitment without effective activation of pro-repair pathways. Such dysregulation likely contributes to persistent, low-grade inflammation and tissue stasis, hallmarks of chronic wounds. These findings are consistent with clinical observations identifying *E. faecalis* as one of the most prevalent isolates in chronic, non-healing wounds [50,51,52]. Mechanistic studies have demonstrated that *E. faecalis* disrupts epithelial and fibroblast signaling, impairs immune regulation, and promotes incomplete epithelial-to-mesenchymal transition, creating a microenvironment conducive to bacterial persistence and impaired tissue regeneration [50,51,52]. The cytokine pattern observed here mirrors these pathogenic effects, emphasizing the role of *E. faecalis* in sustaining chronic inflammatory states and impeding fibroblast-driven repair.

Collectively, these findings reveal that bacterial virulence factors exert divergent, and at times opposing, effects on fibroblast-mediated wound repair. While *S. aureus* and *E. faecalis* impair migration and promote sustained inflammation, *S. pyogenes* and *L. plantarum* appear to support repair through balanced immunomodulation, and *P. aeruginosa* exhibits a context-dependent influence that may vary across strains and environmental conditions. This complexity underscores that bacterial presence alone does not equate to pathogenicity; instead, wound outcomes depend on the equilibrium between deleterious and beneficial microbial activities. The observation that certain species can mitigate the inhibitory actions of others, as seen with *S. pyogenes*, further supports the concept of microbial cross-talk as a determinant of healing trajectories. Understanding these inter-bacterial interactions could inform new therapeutic strategies aimed at leveraging beneficial microbial factors to counteract pathogenic influences.

Several limitations should be acknowledged. The bacterial strains used in this study were ATCC reference isolates, which, while standardized and well-characterized, may not fully capture the genetic heterogeneity, virulence potential, and antibiotic resistance of clinical isolates. In addition, bacterial culture supernatants were employed as proxies for virulence factor–containing secretomes. While this approach allows for the assessment of the collective effect of bacterial secreted products on fibroblast function, it does not provide quantitative control over individual virulence components or their relative abundance. The concentration of virulence factors in culture supernatants can vary depending on bacterial growth phase, medium composition, and culture conditions, and therefore may not precisely reflect in vivo levels. Moreover, the bacterial secretomes analyzed represent complex mixtures of secreted proteins, metabolites, and extracellular vesicles rather than purified virulence components. Although this approach more closely reflects the in vivo wound milieu, it precludes definitive attribution of observed effects to specific molecular entities. Future studies employing proteomic characterization, purification of individual virulence factors, and in vivo validation are necessary to delineate the specific effectors that modulate fibroblast behavior and wound outcomes. Furthermore, wound healing is an inherently multicellular process involving coordinated signaling among fibroblasts, keratinocytes, immune cells, and vascular elements. While the fibroblast model used here provides valuable mechanistic insight, it does not fully recapitulate the cellular and biochemical complexity of the in vivo wound environment. Advanced co-culture and organotypic models will be essential to elucidate how bacterial communities collectively influence the transition from acute to chronic healing.

In summary, this work provides comprehensive mechanistic evidence that bacterial virulence factors differentially regulate fibroblast-driven wound repair through distinct inflammatory and signaling pathways. By dissecting strain-specific effects, we demonstrate that certain bacteria act as potent inhibitors, others exert neutral or even reparative influences, and some interact synergistically to shape the overall healing trajectory. These insights clarify why the mere detection of bacteria in wounds does not necessarily predict poor outcomes and instead highlight the importance of microbial composition, virulence potential, and host context. Collectively, the findings support a paradigm shift toward precision microbiome-based therapeutics aimed at modulating bacterial virulence and harnessing beneficial microbial interactions to restore tissue homeostasis and accelerate wound healing.

## 4. Materials and Methods

### 4.1. Biological Material

#### 4.1.1. Bacterial Strains, and Culture

Five skin-associated bacterial strains were used, each obtained as KIWIK STIK preparations from the American Type Culture Collection (ATCC) (Microbiologics, St Cloud, MN, USA): Lactobacillus plantarum (ATCC 8014), *Enterococcus faecalis* (ATCC 7080), *Staphylococcus aureus* (ATCC BAA-976), *Pseudomonas aeruginosa* (ATCC BAA-1744), and *Streptococcus pyogenes* (ATCC 19615). Quadrant streaking was performed on blood agar plates to obtain pure colonies, followed by incubation at 37 °C for 24 h. A single colony from each strain was inoculated into Lysogeny Broth (B; catalog #610084, MilliporeSigma, Burlington, MA, USA) and cultured at 37 °C with shaking at 200 rpm for 24 h to promote robust growth under aerobic conditions. Each bacterial strain was cultured twice independently in LB at 37 °C with shaking at 200 rpm for 24 h under aerobic conditions. Each strain was cultured twice independently under identical conditions to ensure reproducibility. After incubation, culture supernatants were collected, filtered to remove residual cells, and pooled in equal volumes to generate standardized virulence factor preparations used in all subsequent experiments.

#### 4.1.2. Preparation of Bacterial Supernatants

Bacterial growth was quantified spectrophotometrically at 600 nm (OD_600_). Cultures were centrifuged at 15,000 rpm for 30 min at 4 °C, and the resulting supernatants—containing secreted virulence factors and other extracellular proteins—were passed through 0.2 µm syringe filters (Fisherbrand, catalog #15206869, Thermo Fisher Scientific, Waltham, MA, USA) to remove residual cells. Filtered supernatants were aliquoted into sterile tubes and stored at −80 °C until use.

#### 4.1.3. Total Protein Quantification

A fraction of each filtered bacterial supernatant was used for total protein quantification using the Bicinchoninic Acid Protein Assay (BCA; Thermo Scientific, Waltham, MA, USA) according to the manufacturer’s protocol. Absorbance at 562 nm was measured using a Multiskan Sky spectrophotometer (Thermo Fisher Scientific, Waltham, MA, USA). Protein concentrations were calculated from a standard curve generated using Bovine Serum Albumin (BSA) standards.

#### 4.1.4. Preparation of Gradient Virulence Factor Concentrations

Based on total protein quantification, virulence factor preparations from each bacterial species were diluted to final concentrations of 0.05, 1, 3, 12, and 16 µg/mL. These standardized preparations were used to evaluate their dose-dependent effects on cell viability and wound healing responses in Human dermal fibroblast-neonatal (HDF-n) cells.

### 4.2. In Vitro Cellular Responses to Bacterial Virulence Factors

#### 4.2.1. Cell Culture

The Human dermal fibroblast-neonatal (HDF-n) cell line was obtained from Thermo Fisher Scientific, USA (Catalog #C0045C). Cells were cultured in Medium 106 (M106500) supplemented with Low Serum Growth Supplement (LSGS; catalog #S003K, Gibco™, Catalog #S003K, Thermo Fisher Scientific, Waltham, MA, USA). Cultures were maintained in a humidified incubator at 37 °C with 5% CO_2_. Upon reaching 80–90% confluency, cells were harvested using Trypsin, counted, and seeded at appropriate densities for subsequent assays.

#### 4.2.2. Cellular Viability Assay

HDF-n cells were seeded in 96-well plates at a density of 11,000 cells/well and allowed to attach overnight. Cells were treated with bacterial virulence factor preparations for 24 and 48 h, after which viability was measured using the AlamarBlue assay (Invitrogen #DAL1100, Waltham, MA, USA) according to the manufacturer’s instructions and a previously validated protocol [53]. Fluorescence was measured at 560 nm excitation and 590 nm emission using a Synergy H1 Microplate Reader. Viability was expressed as a percentage relative to untreated control cells. Results are reported as mean ± SEM from three independent experiments.

### 4.3. Wound Healing (Scratch) Assay

HDF-n cells were cultured in 24-well plates until confluent. A scratch was introduced using a sterile 200 µL pipette tip to create a wound-like gap in the monolayer. Detached cells were gently removed with Phosphate-Buffered Saline (PBS). Cells were treated with bacterial virulence factor preparations at 3, 5, and 12 µg/mL, while control wells received fresh medium only. Wound closure was imaged at 0, 24, and 48 h post-injury using an inverted light microscope. The wound area was quantified using ImageJ software version 1.54f, and wound closure was calculated as the percentage reduction in wound area relative to the initial scratch.

### 4.4. Analysis of Cytokine Release Following Bacterial Virulence Factor Exposure

To evaluate the effects of bacterial virulence factors on cytokine release and to indirectly assess their impact on fibroblast migration, proliferation, and angiogenic potential, HDF-n cells were seeded at 66,000 cells/well in 24-well plates. Upon reaching >80% confluency, a linear scratch was introduced into the monolayer using a sterile 200 µL pipette tip to activate the cells and stimulate cytokine secretion. Bacterial virulence factor preparations at 5 µg/mL and 12 µg/mL were then applied to the scratched monolayers. Control groups included untreated scratched and unscratched fibroblast monolayers.

After 48 h of treatment, cell culture supernatants were collected, centrifuged at 1400 rpm for 10 min to remove debris, and 150 µL of each clarified supernatant was used for cytokine analysis. Cytokine and chemokine levels were quantified using the Inflammation 20-Plex Human ProcartaPlex Panel (Catalog #EPX200-12185-901, Thermo Fisher Scientific, Waltham, MA, USA), which measures 20 inflammatory markers including GM-CSF, IFN-α, IL-1β, and E-selectin. Quantification was performed on a LABScan 3D Multiplex System (Luminex Corporation, Austin, TX, USA) based on xMAP® technology.

Samples were processed according to the manufacturer’s instructions, which involved bead vortexing, incubation with detection antibodies, washing, and incubation with streptavidin–phycoerythrin (SAPE), followed by the addition of reading buffer before analysis. Cytokine concentrations were calculated from standard curves and averaged from two independent Luminex readings, following a previously reported protocol [53].

### 4.5. Statistical Analysis

All experiments were performed at least in duplicate to ensure reproducibility. Data are presented as mean ± standard error of the mean (SEM) from independent experiments. Statistical significance was assessed using two-way analysis of variance (ANOVA) followed by Fisher’s least significant difference test for multiple comparisons. A *p*-value < 0.05 was considered statistically significant. All statistical analyses and graphical presentations were conducted using GraphPad Prism version 5 (GraphPad Software, San Diego, CA, USA).

## 5. Conclusions

In this study, we effectively investigated the impact of bacterial virulence factors from seven bacterial strains—*S. aureus*, *P. aeruginosa*, *S. pyogenes*, *E. faecalis*, and *L. plantarum*—characterizing their effects on cell viability, wound healing, and inflammatory markers. Our findings confirm the diverse and variable influences of different bacterial virulence factors on wound healing processes and their modulation of inflammatory responses. Notably, the reversal of delayed wound healing in models treated with *S. aureus* when supplemented with virulence factors from *P. aeruginosa*, *L. plantarum*, or *S. pyogenes* is significant, underscoring the complex interplay between bacterial components in wound management. Additionally, the observed enhancement of wound healing in models treated with *E. faecalis* upon the addition of *S. pyogenes* virulence factors suggests a potential therapeutic benefit, warranting further investigation into its mechanisms and applications in clinical settings. For future studies, we recommend expanding the range of concentrations tested to gain a more detailed understanding of the dose-dependent effects of bacterial virulence factors. Moreover, exploring the combined impacts of multiple bacterial virulence factors would provide a more realistic approximation of their synergistic or antagonistic behaviors in actual wound environments, thereby enhancing the translational relevance of the research.

## Data Availability

The additional data supporting the manuscript are available from the corresponding author upon request.

## Supplementary Materials

The following supporting information can be downloaded at: https://www.mdpi.com/article/10.3390/ijms262110472/s1.
