# Supplementary material for: In Vitro Analysis of the Dynamic Role of the Bacterial Virulence Factors in Skin Wound Healing
Source: Int J Mol Sci. 2025 Oct 28;26(21):10472. doi: 10.3390/ijms262110472 (PMC12607956; doi:10.3390/ijms262110472)
Supplement: Supplementary file 1 [file ijms-26-10472-s001.zip › ijms-3926071-supplementary.pdf]

Supplementary Materials:

Table 1: Optical Density of Bacterial Cultures Measured at 600 nm

Bacteria	Optical Density (OD) at 600 nm
Pseudomonas aeruginosa	1.747
Staphylococcus aureus	1.284
Streptococcus pyogenes	0.444
Staphylococcus epidermidis	1.662
Lactobacillus plantarum	0.105
Enterococcus faecalis	0.804
Escherichia coli	1.556

Table 2: Protein Concentration of Virulence Factors in Bacterial Supernatants

Bacterial Strain	Concentration (µg/ml)
Pseudomonas aeruginosa	1653.7143
Staphylococcus aureus	1870.3810
Streptococcus pyogenes	1500.8571
Staphylococcus epidermidis	1705.1429
Lactobacillus plantarum	1600.3810
Enterococcus faecalis	1923.7143
Escherichia coli	1717.4

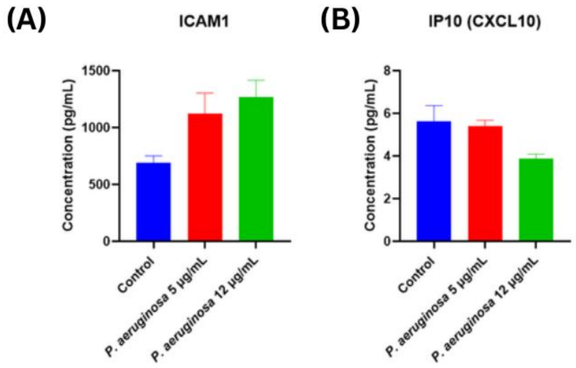

Supplementary Figure 1. Effects of *Pseudomonas aeruginosa* Virulence Factor Supernatants on Fibroblast Inflammatory Mediator Secretion. HDF-n cells were treated with *P. aeruginosa* supernatants at 5 and 12 µg/mL for 48 h. Levels of (A) ICAM1 and (B) IP10 (CXCL10) were measured by multiplex ELISA. Data are presented as mean ± SEM from two independent experiments.

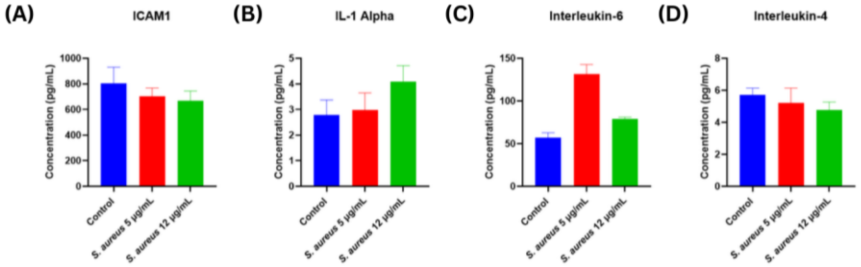

Supplementary Figure 2. Effects of *Staphylococcus aureus* Virulence Factor Supernatants on Fibroblast Inflammatory Mediator Secretion. HDF-n cells were treated with *S. aureus* supernatants at 5 and 12 µg/mL for 48 h. Concentrations of (A) ICAM1, (B) IL-1α, (C) IL-6, and (D) IL-4 were quantified by multiplex ELISA. Data are presented as mean ± SEM from two independent experiments.

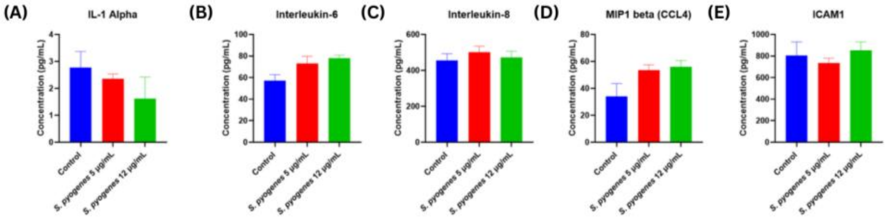

Supplementary Figure 3. Effects of *Streptococcus pyogenes* Virulence Factor Supernatants on Fibroblast Inflammatory Mediator Secretion. HDF-n cells were treated with *S. pyogenes* supernatants at 5 and 12 µg/mL for 48 h. Concentrations of (A) IL-1α, (B) IL-6, (C) IL-8, (D) MIP-1β (CCL4), and (E) ICAM1 were quantified by multiplex ELISA. Data are presented as mean ± SEM from two independent experiments.

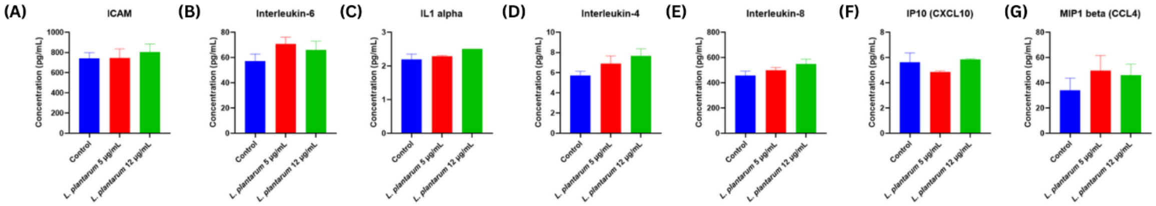

Supplementary Figure 4. Effects of *Lactobacillus plantarum* Virulence Factor Supernatants on Fibroblast Inflammatory Mediator Secretion. HDF-n cells were treated with *L. plantarum* supernatants at 5 and 12 µg/mL for 48 h. Concentrations of (A) ICAM1, (B) IL-6, (C) IL-1α, (D) IL-4, (E) IL-8, (F) IP10 (CXCL10), and (G) MIP-1β (CCL4) were quantified by multiplex ELISA. Data are presented as mean ± SEM from two independent experiments.

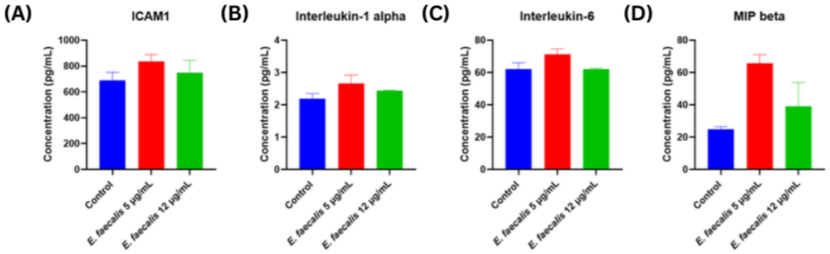

Supplementary Figure 5. Effects of *Enterococcus faecalis* Virulence Factor Supernatants on Fibroblast Inflammatory Mediator Secretion. HDF-n cells were treated with *E. faecalis* supernatants at 5 and 12 μg/mL for 48 h. Concentrations of (A) ICAM1, (B) IL-1α, (C) IL-6, and (D) MIP-1β were quantified by multiplex ELISA. Data are presented as mean ± SEM from two independent experiments.
